# A Red Cross Society

**Published:** 1904-11-26

**Authors:** 


### The Hospital.

A Journal of

The Medical Sciences and Hospital Administration.

Vol. XXXVII.—No. 948. NOVEMBER 26, 1904.

### A Red Cross Society.

A circular letter was published on Monday, calling attention to the want, in the British Empire, of any effective organisation for rendering aid to the sick and wounded in time of war, and suggesting a method by which this deficiency might be supplied. It will be remembered that the Geneva Convention of 1864 submitted for the acceptance of the powers a series of regulations to be thenceforward binding upon belligerents, and which had for their chief object to secure the neutrality of the sick and wounded and of those engaged in attendance upon them; the red cross on a white ground being suggested as a distinctive badge for the purpose of indicating the persons and establishments thus neutralised, and being either borne as a flag upon ambulances or hospitals or worn as an armlet by properly authorised individuals. The regulations in question, which were somewhat amplified by a second convention in 1868, were speedily adopted by every European Power, by Japan, and by Persia; and their adoption, by reason of the comparative security which it afforded to the persons and property of those engaged in tending the sick, at once opened the door for a large and active display of benevolence in this direction. Every civilised country has at one time or another contributed under the "Red Cross" to diminish the unavoidable suffering incidental to war; and almost every country has formed a definite organisation, or "Red Cross Society," not only for the assistance of its own naval or military forces in case of need, but also as a means of extending charitable help to belligerents of other nations. In England alone, or almost alone, this has not been done; and Red Cross work, on every occasion of its having been called for, has been hastily undertaken by extemporised methods. It is now proposed to supersede these extemporised methods, and to obviate any recurrence of the waste and confusion which have been their inevitable concomitants, by the establishment of an Imperial Red Cross Society on a well-considered basis, capable of speedily gathering up and of applying the scattered threads of individual activity and benevolence, and of entering into definite relations with the Admiralty, the War Office, and with naval or military commanders. The Red Cross Society of Japan was described in the "Encyclopædia Britannica," two years ago, as having an income of 2,000,000 yen annually, a fine hospital in Tokio, a large staff of trained nurses of both sexes, and two hospital ships specially built and equipped for the transport of wounded men. Their arrangements were so excellent that, during the early part of the campaign in Pechili in 1900, the French column entrusted its wounded to the care of the Japanese. In the present war the society has been largely instrumental in bringing about the excellence of personal and camp equipment to which so many correspondents have borne testimony and of which the use of aluminium for the soldiers' water-bottles is a single but noteworthy illustration.

In this country a national society for aid to the wounded in war was brought into existence in 1870 for the purpose of rendering assistance to the French and Germans, and it has been called into renewed activity on subsequent occasions, especially, of course, in order to meet our own requirements in South Africa. With the termination of an emergency it has been suffered to fall into a state of suspended animation, discharging no functions, and represented only by a general council, a sort of nucleus around which, in case of need, its forces might be once more arrayed. Of this nucleus, her Majesty the Queen is President, and Viscount Knutsford is Chairman; and the object of the letter to which we now desire to call attention is to convert the nucleus into an active Red Cross Society, with branches in all parts of the Empire, and with an organisation so constructed as to admit of being brought into immediate and active operation, without overlapping, and without waste either of time, of material, or of money. It is proposed that the society should contain representatives of the existing council, of the St. John's Ambulance Association, by which more than 2,000 bearers were sent to South Africa during the war, of the St. Andrew's Ambulance Association of Scotland, of the Admiralty, and of the War Office; so that it would be in a position to know the probable wants of any expeditionary force despatched by this country, and should also be in a position to meet these wants with promptitude and completeness by its relations with the several local or branch societies, with whose powers and resources it would be acquainted. It is estimated that although a considerable income might be usefully expended in the construction and development of the necessary organisation, yet that expenditure of this kind would not be required for more than a short period; and that, when the society was once complete, its mere maintenance would be a comparatively inexpensive matter. The basis of membership now proposed is either a single donation of not less than five pounds, or an annual subscription of not less than five shillings; while the local expenses of branches would be covered by a very trifling sum. The general idea is that the central society should be able at once to indent upon its branches for such requirements as they had previously announced their ability and their readiness to supply, so that in this way everything which was wanted would be asked for from those who were prepared for the demand, and nothing that was superfluous would be either asked for or received. The St. John's and St. Andrew's Ambulance Associations would be called upon for bearers, the hospital reserves for nurses, the branch formed in a great seaport for a hospital ship, the branch formed by a great railway company for a hospital train, and so forth; while, if woollen nightcaps or warm clothing were in demand, they would be obtained from the lady members of the branches in provincial towns. In such an organisation there would be a place for every kind of effort, and for every amount and kind of contribution, from the least to the greatest; and, if it can be carried into effect in a manner equal to the desires of its promoters, it will be calculated to wipe away, at least in one direction, the reproach of unreadiness which England has so often incurred, and to save her sailors and soldiers from much unnecessary suffering.

